# Synthesis of RuO_2_-Co_3_O_4_ Composite for Efficient Electrocatalytic Oxygen Evolution Reaction

**DOI:** 10.3390/nano15171356

**Published:** 2025-09-03

**Authors:** Jingchao Zhang, Yingping Bu, Jia Hao, Wenjun Zhang, Yao Xiao, Naihui Zhao, Renchun Zhang, Daojun Zhang

**Affiliations:** 1 Henan Key Laboratory of New Optoelectronic Functional Materials, College of Chemistry and Chemical Engineering, Anyang Normal University, Anyang 455000, China; 2 College of Chemistry, Zhengzhou University, Zhengzhou 450001, China

**Keywords:** oxygen evolution reaction, ruthenium oxide, cobalt oxide, RuO_2_-Co_3_O_4_ composite

## Abstract

Among various H_2_ production methods, splitting water using renewable electricity for H_2_ production is regarded as a promising approach due to its high efficiency and zero carbon emissions. The oxygen evolution reaction (OER) is an important part of splitting water, but also the main bottleneck. The anodic oxygen evolution reaction (OER) for water electrolysis technology involves multi-electron/proton transfer and has sluggish reaction kinetics, which is the key obstacle to the overall efficiency of electrolyzing water. Therefore, it is necessary to develop highly efficient and cheap OER electrocatalysts to drive overall water splitting. Herein, a series of efficient RuO_2_-Co_3_O_4_ composites were synthesized via a straightforward three-step process comprising solvothermal synthesis, ion exchange, and calcination. The results indicate that using 10 mg of RuCl_3_·xH_2_O and 15 mg of Co-MOF precursor in the second ion exchange step is the most effective way to acquire the Co_3_O_4_-RuO_2_-10 (RCO-10) composite with the largest specific area and the best electrocatalytic performance after the calcination process. The optimal Co_3_O_4_-RuO_2_-10 composite powder catalyst displays low overpotential (*η*_10_ = 272 mV), a small Tafel slope (64.64 mV dec^−1^), and good electrochemical stability in alkaline electrolyte; the overall performance of Co_3_O_4_-RuO_2_-10 surpasses that of many related cobalt-based oxide catalysts. Furthermore, through integration with a carbon cloth substrate, Co_3_O_4_-RuO_2_-10/CC can be directly used as a self-supporting electrode with high stability. This work presents a straightforward method to design Co_3_O_4_-RuO_2_ composite array catalysts for high-performance electrocatalytic OER performance.

## 1. Introduction

Among various H_2_ production methods, using renewable electricity for green H_2_ production is considered a promising approach due to its high efficiency and zero carbon emissions [1,2,3,4,5,6,7]. During the process of electrolyzing water, the anodic oxygen evolution reaction (OER) forms the main energy barrier, which is much higher than that of the cathode hydrogen evolution reaction (HER) [8,9,10,11,12]. Notably, OER possesses sluggish reaction kinetics and involves multi-electron/proton transfer and is the key obstacle to the overall efficiency of electrolyzing water [13,14,15,16,17,18]. Exploring OER catalysts with high activity and stability can provide an effective solution to these problems [19,20]. Hence, it is necessary to develop low-cost and high-performance OER electrocatalysts to drive overall water splitting.

At present, the precious metal oxides IrO_2_ and RuO_2_ are considered benchmark OER catalysts due to their high activity over the entire pH range [21,22,23]. However, noble metal Ir-based catalysts cannot be widely applied in the industrial field due to their prohibitive cost and limited resources [24,25,26]. Therefore, it is urgently necessary to explore new and efficient non-noble metal or low-content noble metal catalysts with relatively low cost. Among the platinum group elements, ruthenium is widely used as the cheapest noble metal [27,28]. Considering that Ru costs approximately one-sixth the price of Ir and has better intrinsic activity, there are great research prospects for Ru-based nanomaterials in acidic or alkaline water electrolyzers. However, the stability of RuO_2_ is not satisfactory in either acidic or alkaline electrolytes due to the inevitable corrosion and dissolution in the form of high-valence Ru^n>4+^ species. Recently, Du et al. reported that the design of interface engineering of RuO_2_/CoO_x_ can stabilize RuO_2_ [29]. Jiang’s group explored a Ni_m_Ru_n_O_x_-C catalyst with excessive nickel (m > n), which can protect Ru and exhibit stable performance for OER [30]. Thus, it can be predicted that immobilizing Ru species in transition-metal oxide matrices can greatly improve the stability of RuO_2_.

Transition-metal oxide (TMO) electrocatalysts have attracted much attention due to their advantages of abundant reserves, low cost, and robust electrochemical performance in an alkaline medium [31,32,33,34,35,36,37]. In particular, the spinel Co_3_O_4_ structure of cobalt oxide catalysts consists of two types of Co sites and multiple oxidation valent states; Co_3_O_4_ micro-nanostructure catalysts show moderate adsorption energies for OER intermediates and exhibit high OER catalytic activities in alkaline electrolytes [38,39]. Moreover, the OER properties of Co_3_O_4_ can be regulated via the incorporation of secondary noble metal oxides [40,41,42]. According to previous reports, doping noble metal materials into cobalt-based oxides can alter the intrinsic electronic structure of catalysts, thereby improving catalytic activity [43,44,45,46,47]. In addition, cobalt oxides derived from metal-organic framework (MOF) precursors play a crucial role in electrocatalytic fields such as oxygen reduction reaction (ORR) and oxygen evolution reaction (OER) due to their large specific surface area and porosity [48,49,50,51,52,53].

In this work, MOF-derived RuO_2_-Co_3_O_4_ (RCO) powder and its array catalyst were fabricated using a simple three-step synthesis method, and their electrocatalytic OER performance was characterized. The optimal RuO_2_-Co_3_O_4_-10 sample not only has a stable structure but also exhibits a large BET specific surface area (89.20 m^2^g^−1^). The electrocatalytic performance of the RuO_2_-Co_3_O_4_-10 was tested by placing the sample in 1 M KOH solution: it exhibited low overpotential (*η*_10_ = 272 mV) and small Tafel slope (64.64 mV dec^−1^). The stability of the electrode material was tested using the constant current method, and the potential change in the RuO_2_-Co_3_O_4_-10-modified electrode was negligible during the stability test, which lasted for 5 h. To overcome the limitation of the long-term stability test, Co_3_O_4_/RuO_2_-10 composite was grown on carbon cloth (RCO-10/CC) using the same method. Compared to the Co_3_O_4_/RuO_2_-10 sample, the as-synthesized RuO_2_-Co_3_O_4_-10/CC electrode exhibits a lower overpotential of 262 mV and demonstrates outstanding long-term durability over 24 h. This work presents a straightforward method to design RuO_2_-Co_3_O_4_ composite array catalysts for high-performance electrocatalytic OER performance.

## 2. Experimental Section

### 2.1. Chemicals

Co(NO_3_)_2_·6H_2_O, C_16_H_36_BrN, CO(NH_2_)_2_, ethanol, and potassium hydroxide (KOH) were purchased from Sinopharm Chemical Reagent Co., Ltd. (Shanghai, China); 1,3,5-benzenetricarboxylate, RuCl_3_·xH_2_O, and Nafion were obtained from Aladdin Industrial Corporation (Shanghai, China). In this work, all chemicals were AR grade and directly used without further purification.

### 2.2. Preparation of Samples

Synthesis of Co-MOF powder precursor: Firstly, 1.2 g of urea, 0.1 g of tetrabutylammonium bromide, 0.1 mmol of Co(NO_3_)_2_·6H_2_O, and 0.1 mmol of 1,3,5-benzenetricarboxylate were sequentially added to a 20 mL reaction vessel liner and dispersed in a mixed solution of 10 mL of H_2_O and 5 mL of 75% ethanol. The reaction mixture was stirred for 40 min until the solution became transparent. Afterwards, the reaction kettle was placed in an oven at 100 °C and maintained for 12 h. Then, the obtained product was washed three times with water and ethanol, then Co-MOF precursors were collected by centrifugation and dried under vacuum conditions.

Synthesis of RuCo-MOFs powder: 15 mg of Co-MOF was separately dispersed in a mixed solution of 10 mL H_2_O and 5 mL 75% ethanol containing 5, 10, or 15 mg of RuCl_3_·xH_2_O with vigorous stirring. An hour later, the brownish black suspension was centrifuged and dried in an oven at 60 °C to obtain RuCo-MOF-5, RuCo-MOF-10, and RuCo-MOF-15.

Preparation of RuO_2_-Co_3_O_4_ composite powder sample: RuCo-MOF-10 precursor was heated to 350 °C in a furnace at a rate of 2 °C min^−1^ and then kept for 2 h to obtain the final RuO_2_-Co_3_O_4_-10 (RCO-10) sample. Under the same calcination conditions, Co-MOF, RuCo-MOF-5, and RuCo-MOF-15 precursors could yield the products Co_3_O_4_ (CO), RuO_2_-Co_3_O_4_-5 (RCO-5), and RuO_2_-Co_3_O_4_-15 (RCO-15), respectively.

Preparation of Co_3_O_4_-RuO_2_-10/CC composite self-supported electrode: A piece of hydrophilic carbon cloth (CC) of 1 cm × 2.5 cm was separately rinsed in a 3 M HCl solution, deionized water, and ethanol. Initially, Co-MOF/CC was obtained with the same synthesis step used for the Co-MOF powder precursor, except that the carbon cloth was added after the reaction mixture was stirred evenly and the reaction conditions remained unchanged. The pink Co-MOF/CC was washed with water and ethanol, then dried at 80 °C for 12 h. The average loading mass is ~4 mg cm^−2^. Subsequently, the RuCo-MOF-10/CC was obtained by immersing Co-MOF/CC in a H_2_O and ethanol (75%) mixture (H_2_O/ethanol = 2:1) containing 6.7 mg RuCl_3_·xH_2_O and just holding for 1 h at room temperature. The brown RuCo-MOF-10/CC was dried and calcined at 350 °C for 2 h to produce the Co_3_O_4_-RuO_2_-10/CC composite self-supporting electrocatalyst.

### 2.3. Material Characterization

High-resolution field-emission scanning electron microscopy (FE-SEM, Hitachi SU8010, Tokyo, Japan) and field-emission transmission electron microscopy (TEM, Tecnai G2S Twin F20, FEI, Hillsboro, OR, USA) were used to analyze the morphology and structure of the synthesized samples. The crystal structure and phase composition of the samples were all studied on an X-ray powder diffractometer (XRD, PANalytical X’ Pert operated at 40 kV and 40 mA) with a step size of 0.05252° and 194 s per step. The chemical valence state and surface elemental composition of the samples were analyzed via X-ray photoelectron spectroscopy (XPS, Thermo Scientific K-Alpha, McMurdo, Antarctica, USA). The elemental distribution of the sample was characterized on a X-ray energy spectrometer (EDX, XFlash Detector, Bruker, Berlin, Germany). The specific surface area and pore size of the samples were determined using a fully automated rapid specific surface area and porosity analyzer (BET, Gemini VII 2390, Micromeritics Instrument Corporation, Norcross, GA, USA).

### 2.4. Electrode Preparation and Electrochemical Measurements

All electrochemical measurements were conducted on an electrochemical workstation (CHI 760E, Chenhua, Shanghai, China) using a three-electrode system at room temperature. The Hg/HgO electrode is used as the reference electrode, platinum wire is used as the counter electrode, and the glassy carbon electrode (0.196 cm^2^) of the rotating disk electrode (RDE) is used as the working electrode. The working electrode was prepared as follows: 5 mg of catalyst was dispersed in a mixed solution of 700 μL distilled H_2_O, 250 μL isopropanol, and 50 μL Nafion (5 wt%), and the mixed solution was sonicated for 30 min to obtain a uniform ink. Then, we used a pipette to take 10 μL of ink and evenly drop it onto the surface of the dry RDE.

For the OER tests, the powder samples were placed in alkaline electrolyte (1 M KOH solution). Before electrochemical testing, O_2_ needs to be introduced into KOH solution for at least 30 min to ensure that the electrolyte is in an oxygen-saturated state [54]. Firstly, 20 cycles of cyclic voltammetry curves (CVs) were performed at a scan rate of 20 mV s^−1^ and a voltage range of 0–0.3 V (vs. Hg/HgO) to stabilize the electrode surface. Afterwards, linear sweep voltammetry (LSV) curves and Tafel plot tests were performed at a scan rate of 5 mV s^−1^. The Tafel slope values were obtained by fitting the Tafel plot curves via the equation *η* = a + b log *j*, where *η*, *j*, and b correspond to the overpotential, current density, and Tafel slope, respectively [55]. CV measurements were conducted at different scan rates (10–100 mV s^−1^) in the non-Faraday region with a voltage range of 0–0.1 V (vs. Hg/HgO) to determine the *C*_dl_ value of the synthesized sample and evaluate the ECSA. Electrochemical impedance spectroscopy (EIS) is tested under open circuit voltage with a range of 0.1–10^6^ Hz. The stability of the sample was evaluated using the constant current method. For the Co_3_O_4_-RuO_2_-10/CC self-standing electrode, the geometrical area immersed in electrolyte is 1 cm^2^. During the testing process, *iR* compensation was not performed and all tests were conducted at room temperature [56]. All potentials are converted vs. RHE according to the formula *E*_RHE_ = *E*_Hg/HgO_ + 0.098 + 0.059 × pH.

## 3. Results and Discussion

**Figure 1:**
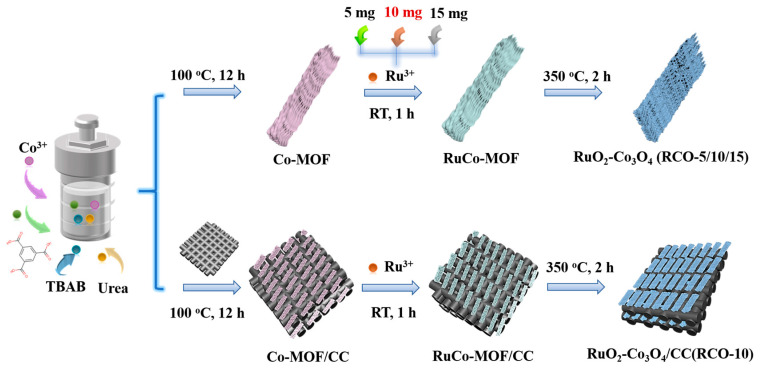
Schematic diagram of the synthesis of Co_3_O_4_-RuO_2_ series powder samples and Co_3_O_4_-RuO_2_-10/CC integrated electrode.

The synthesis route of the RuO_2_-Co_3_O_4_ composite sample is shown in Figure 1. RuO_2_-Co_3_O_4_ layered arrays were successfully prepared using a straightforward three-step method. Firstly, metal/organic framework (MOF) precursors were synthesized using the solvothermal method at 100 °C for 12 h. Afterwards, the precursor powder was added to a mixed RuCl_3_·xH_2_O solution of H_2_O and ethanol, and stirred thoroughly at room temperature for 1 h to introduce Ru^3+^ into the MOF precursors. Finally, it was calcined at 350 °C for 2 h to obtain the target product. The Co_3_O_4_-RuO_2_-10/CC integrated electrode was synthesized using the same approach, except that carbon cloth was added in the first step.

**Figure 2:**
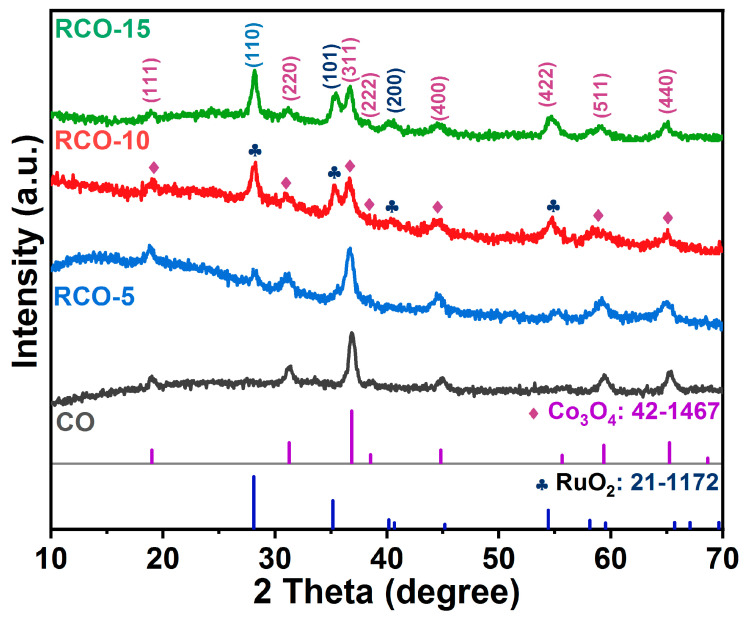
PXRD patterns of Co_3_O_4_, Co_3_O_4_-RuO_2_-5, Co_3_O_4_-RuO_2_-10, and Co_3_O_4_-RuO_2_-15 samples.

The phase composition and crystal structure of the sample were analyzed using PXRD. Figure 2 shows the PXRD and JCPDS standard patterns of the Co_3_O_4_ and Co_3_O_4_-RuO_2_ series samples. The diffraction peaks of Co_3_O_4_, Co_3_O_4_-RuO_2_-5, Co_3_O_4_-RuO_2_-10, and Co_3_O_4_-RuO_2_-15 at 19.0°, 31.3°, 36.8°, 38.5°, 44.8°, 55.6°, 59.3°, and 65.2° are attributed to the (111), (220), (311), (222), (400), (422), (511), and (440) crystal planes of Co_3_O_4_ (JCPDS No.: 42-1467). The diffraction peaks of RCO-5, RCO-10, and RCO-15 at 28.1°, 35.2°, and 40.2° are attributed to the (110), (101), and (200) crystal planes of RuO_2_ (JCPDS No.: 21-1172), respectively [57,58]. It can be clearly observed that the diffraction peak of sample Co_3_O_4_ (CO) matches pure phase Co_3_O_4_, while the series of samples RCO-5, RCO-10, and RCO-15 containing the Ru element exhibit both Co_3_O_4_ and RuO_2_ diffraction peaks. Furthermore, both Co_3_O_4_ and RuO_2_ diffraction peaks were broad, suggesting the presence of small nanoparticles in the composite. The following pattern can also be observed: as the content of added Ru^3+^ ion gradually increases, the diffraction peaks corresponding to Co_3_O_4_ show a gradual weakening, while the diffraction peaks of RuO_2_ show a strengthened trend. This indicates the successful introduction of Ru^3+^ ions into the MOF precursors.

**Figure 3:**
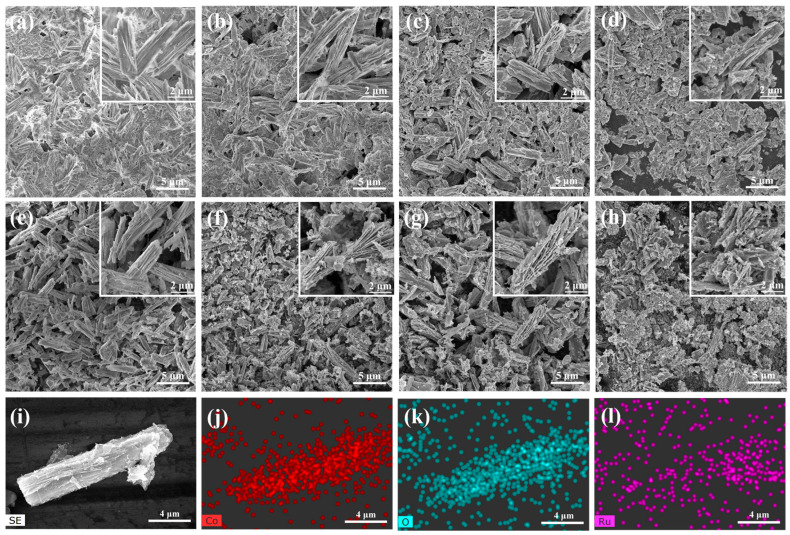
(**a**–**d**) SEM images of precursors of Co-MOF, RuCo-MOF-5, RuCo-MOF-10, and RuCo-MOF-15; (**e**–**h**) SEM images of as-prepared Co_3_O_4_, Co_3_O_4_-RuO_2_-5, Co_3_O_4_-RuO_2_-10, and Co_3_O_4_-RuO_2_-15 samples; (**i**–**l**) EDX mapping diagram of optimal Co_3_O_4_-RuO_2_-10 sample.

The morphology and structure of the synthesized Co-MOFs were studied by using FE-SEM and XRD (Figure S1). Figure 3a–d show the SEM images of the precursors of Co-MOF, RuCo-MOF-5, RuCo-MOF-10, and RuCo-MOF-15, respectively. The enlarged image in the upper right corner indicates that the morphology of the RuCo-MOF series samples is a layered structure of nanosheets stacked together. The addition of an appropriate RuCl_3_·xH_2_O solution did not significantly influence the morphology, but excessive RuCl_3_·xH_2_O addition can damage the original morphology and structure.

Figure 3e–h show SEM images of Co_3_O_4_, Co_3_O_4_-RuO_2_-5, Co_3_O_4_-RuO_2_-10, and Co_3_O_4_-RuO_2_-15 generated from the precursor after calcination at 350 °C for 2 h. After calcination, a small portion of thin broken nanosheets was observed to adhere to the layered structure, but the overall morphology remained intact. Based on the enlarged SEM image, it can be observed that the nanosheets of Co_3_O_4_ are tightly arranged, while the introduction of Ru^3+^ results in a fan-shaped scattering of the layered structure, which can increase the effective specific surface area. The unique morphology structure is conducive to providing a larger specific surface area with more catalytic active sites and promoting the penetration of the electrolyte in the reaction process. Figure 3i–l show the EDX mapping of Co_3_O_4_-RuO_2_-10, confirming the uniform distribution of Co, Ru, and O elements in the composite sample.

Using EDX to analyze the types and contents of elements for the as-synthesized electrocatalysts. The peak appearing around 1.5 keV is due to the use of an aluminum sample stage, where signal peaks from the substrate are observed during testing in thinner areas of the sample. Figure S2 shows the EDX spectra of all samples. In Figure S2a, the atomic ratio of Co to O is 1:1.48, indicating that the main component of the samples is Co_3_O_4_. The atomic ratio Ru:Co:O of Co_3_O_4_-RuO_2_-5 in Figure S2b is 1:13.27:25.11, indicating the presence of Co_3_O_4_ and RuO_2_ components in the sample, and with a higher content of Co_3_O_4_. The atomic ratio Ru:Co:O of Co_3_O_4_-RuO_2_-10 in Figure S2c is 1:7.24:11.7, indicating the presence of Co_3_O_4_ and RuO_2_ components in the sample, with a moderate amount of RuO_2_. The atomic ratio Ru:Co:O of Co_3_O_4_-RuO_2_-15 in Figure S2d is 1:3.66:7.83, indicating the presence of Co_3_O_4_ and RuO_2_ components in the sample, with a higher content of RuO_2_. All the results are consistent with the PXRD characterization results.

**Figure 4:**
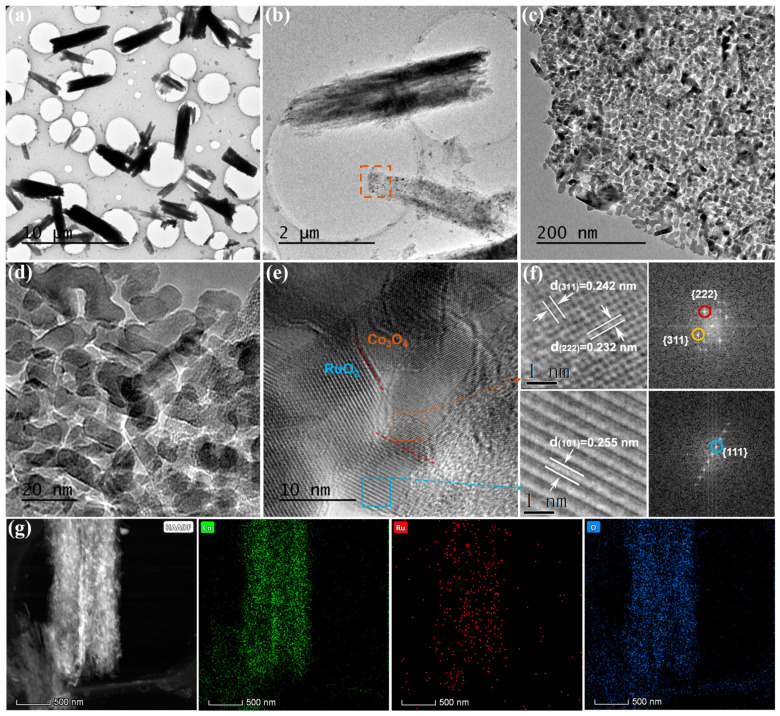
(**a**–**d**) TEM images of Co_3_O_4_-RuO_2_-10 sample; (**e**) HRTEM images of Co_3_O_4_-RuO_2_-10 with Co_3_O_4_ and RuO_2_ interface; (**f**) corresponding magnified dotted square area and FFT in (**e**); (**g**) HAADF and STEM-EDX element maps of Co_3_O_4_-RuO_2_-10 sample.

The TEM images in Figure 4a–c show that the Co_3_O_4_-RuO_2_-10 micro-layers were constructed with ultra-thin nanosheets. Furthermore, as shown in Figure 4d, the sintered sample has high crystallinity and a porous structure. The enlarged HRTEM image in Figure 4e clearly shows the heterointerface around the microcrystalline grain boundaries of Co_3_O_4_ and RuO_2_, highlighted by the red dashed line. The lattice spacing of 0.232 and 0.242 nm could be assigned to the (222) and (311) faces of Co_3_O_4_, whereas the lattice fringes with spacings of 0.255 nm were assigned to the (101) lattice plane of RuO_2_ (Figure 4f). The corresponding fast Fourier transform (FFT) pattern in the right part of Figure 4f confirms the polycrystal nature of Co_3_O_4_ and RuO_2_. Furthermore, the high-angle annular dark field (HAADF) STEM-EDX element maps also confirms the successful synthesis of the Co_3_O_4_-RuO_2_ composite (Figure 4g).

**Figure 5:**
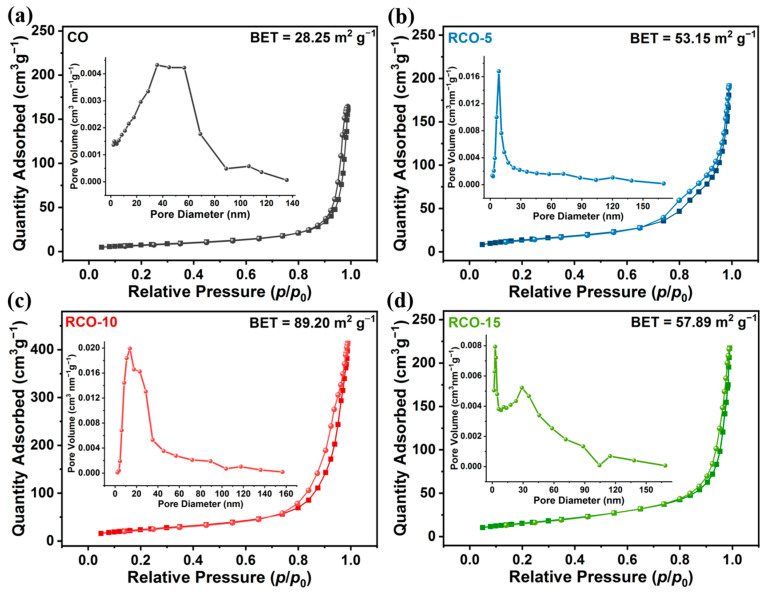
(**a**–**d**) N_2_ adsorption/desorption isotherms of Co_3_O_4_, Co_3_O_4_-RuO_2_-5, Co_3_O_4_-RuO_2_-10, and Co_3_O_4_-RuO_2_-15 samples; the inset illustrates pore size distribution plots.

The specific surface area and pore size of the synthesized samples were measured using N_2_ adsorption/desorption isotherms, indicating the presence of abundant mesoporous structures in the samples. In particular, all the Co_3_O_4_-RuO_2_ series catalysts have higher surface areas than Co_3_O_4_. Figure 5a–d show the Brunauer–Emmett–Teller (BET) plots of Co_3_O_4_, Co_3_O_4_-RuO_2_-5, Co_3_O_4_-RuO_2_-10, and Co_3_O_4_-RuO_2_-15 powder samples with specific surface areas of 28.25, 53.13, 89.20, and 57.89 m^2^g^−1^, respectively. The Co_3_O_4_-RuO_2_-10 exhibits the largest specific surface area among the four samples, which is approximately 3.2 times that of pure Co_3_O_4_. The inset graphs of Figure 5a–d show the pore size distribution of the four samples, and the corresponding pore size distribution was also measured using the Barrett–Joyner–Halenda (BJH) method. The concentrated pore sizes of Co_3_O_4_, Co_3_O_4_-RuO_2_-5, Co_3_O_4_-RuO_2_-10, and Co_3_O_4_-RuO_2_-15 are 35.77, 8.49, 13.79, and 28.89 nm, respectively, indicating that the Co_3_O_4_-RuO_2_-10 sample possesses the highest proportion of mesopores in the as-synthesized samples. The surface areas of the Co_3_O_4_-RuO_2_ series catalysts increased with RuO_2_ loading up to ~12 at.%, then decreased slightly at higher RuO_2_ values.

Due to its large BET specific surface area and mesoporous structure, the Co_3_O_4_-RuO_2_-10 sample is advantageous in providing more active sites and improving charge transport ability, thereby enhancing electrocatalytic OER efficiency.

**Figure 6:**
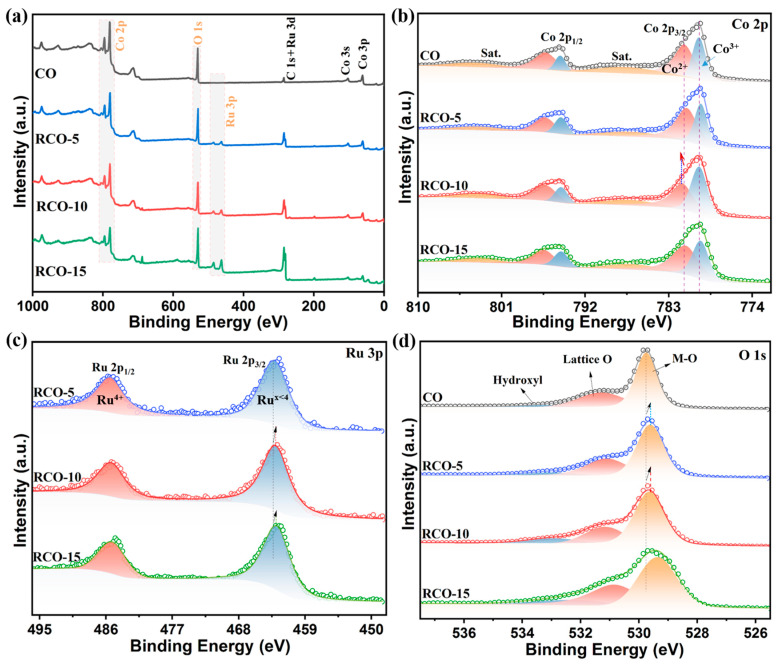
(**a**) XPS survey of Co_3_O_4_, Co_3_O_4_-RuO_2_-5, Co_3_O_4_-RuO_2_-10, and Co_3_O_4_-RuO_2_-15 samples; high-resolution XPS spectra of (**b**) Co 2p, (**c**) Ru 3p, and (**d**) O 1s.

We further used XPS to understand the elemental composition and chemical valence state for the as-prepared samples. Figure 6a shows the survey spectra of the Co_3_O_4_, Co_3_O_4_-RuO_2_-5, Co_3_O_4_-RuO_2_-10, and Co_3_O_4_-RuO_2_-15 samples with the C element used for calibration. It is evident that Co and O elements are present in Co_3_O_4_, while Co, O, and Ru elements are present in Co_3_O_4_-RuO_2_-5, Co_3_O_4_-RuO_2_-10, and Co_3_O_4_-RuO_2_-15 catalysts, confirming the successful introduction of the Ru element into the structure. From the graph, it can also be observed that as the content of added RuCl_3_·xH_2_O increases, the peak of Ru 3p gradually strengthens, which is consistent with the test results of XRD and EDX. Figure 6b shows the high-resolution XPS spectra of Co2p. From Figure 6b, it can be observed that there are two pairs of spin–orbit peaks and one pair of satellite peaks in the Co 2p region of Co_3_O_4_-RuO_2_-10. The two peaks with binding energies of 779.7 and 794.6 eV are attributed to Co 2p_3/2_ and Co 2p_1/2_ of Co^3+^, respectively, while the two peaks with binding energies of 781.5 and 796.3 eV are attributed to Co 2p_3/2_ and Co 2p_1/2_ of Co^2+^, indicating that the Co element exists in a mixed-valence state in the electrocatalyst. In addition, the two peaks with binding energies at 787.2 and 803.4 eV are both satellite peaks (Sat.) [37,38,39]. Compared with other samples, Co_3_O_4_-RuO_2_-10 has the greatest positive shift in the binding energy of Co 2p. This is due to the high electronegativity of the Ru element, which promotes the transfer of electrons from Co_3_O_4_ to RuO_2_ and accumulates more positive charges. The high electronegativity of the Ru element and the interaction between Co and adjacent Ru atoms are key factors in improving the interfacial charge redistribution and can thereby enhance the catalytic activity. Figure 6c shows a high-resolution XPS image of the synthesized Ru 3p sample, with peaks around 463.0 and 485.3 eV attributed to Ru 3p_3/2_ and Ru 3p_1/2_, respectively. The binding energy of 463.3 eV (Ru 3p_3/2_) and 485.6 eV (Ru 3p_1/2_) was attributed to Ru^x<4+^ and Ru^4+^, respectively. There is an obvious binding energy shift to lower energies of the Ru 3p_3/2_ peak in Co_3_O_4_-RuO_2_-10 and Co_3_O_4_-RuO_2_-15 compared to Co_3_O_4_-RuO_2_-5, suggesting that the electron densities of the Ru atomic centers have changed, resulting in electron enrichment on Ru sites and higher content of low-valence ruthenium [29,30,40,59]. Figure 6d shows an O 1s high-resolution XPS image of the synthesized sample; similarly, the O 1s binding energy of the Co_3_O_4_-RuO_2_ series samples also shows a negative shift relative to that of Co_3_O_4_.

The peak with a binding energy around 529.6 eV is attributed to M-O bonds (M=Ru, Co, and Ni), the peak with a binding energy around 531.2 eV is attributed to lattice oxygen, and the peak with a binding energy around 532.9 eV is attributed to adsorbed hydroxyl oxygen [60]. The successful introduction of RuO_2_ and the heterogeneous interfacial synergistic effect are both beneficial for improving the intrinsic OER catalytic performance.

**Figure 7:**
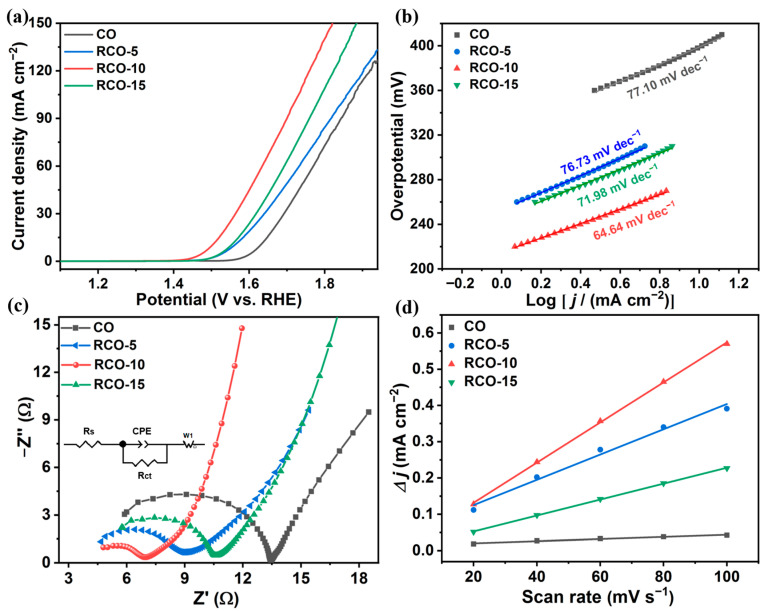
Electrochemical test plots of Co_3_O_4_, Co_3_O_4_-RuO_2_-5, Co_3_O_4_-RuO_2_-10, and Co_3_O_4_-RuO_2_-15: (**a**) LSV curves without iR compensation; (**b**) Tafel slope curves; (**c**) Nyquist plots and the equivalent circuit inset; (**d**) *C*_dl_ value calculated based on the corresponding CV curves.

The OER performance of different samples was investigated in 1 M KOH solution saturated with O_2_ using a standard three-electrode system. In addition, bubbles continuously form on the surface of the catalyst film during the reaction process. To reduce their impact, the rotational speed of the working electrode is always maintained at 1600 r.p.m. during electrochemical testing. Figure 7a shows the polarization curves of all catalysts with a scan rate of 5 mV s^−1^. The Co_3_O_4_-RuO_2_-10 exhibited excellent OER activity, with an overpotential of 272 mV required to reach 10 mA cm^−2^. Co_3_O_4_, Co_3_O_4_-RuO_2_-5, and Co_3_O_4_-RuO_2_-15 require overpotentials of 399, 331, and 322 mV, respectively, to achieve the same current density.

Analyzing the LSV data reveals that compared to pure Co_3_O_4_, the optimized addition of Ru^3+^ during the second ion exchange step leads to 1.5-fold lower overpotential and 40-fold higher current density. The RuO_2_-Co_3_O_4_ composite samples obtained through calcination of the RuCo-MOF precursor significantly enhance the OER kinetics of catalysts due to electron transfer between the two structures. By examining the OER kinetics through the Tafel plots, as shown in Figure 7b, the Tafel slope of Co_3_O_4_-RuO_2_-10 is 64.64 mV dec^−1^, which is lower than Co_3_O_4_ (77.10 mV dec^−1^), Co_3_O_4_-RuO_2_-5 (76.73 mV dec^−1^), and Co_3_O_4_-RuO_2_-15 (71.98 mV dec^−1^), indicating that Co_3_O_4_-RuO_2_-10 has the fastest OER kinetics among the four samples. Usually, electrochemical impedance spectroscopy (EIS) is used to evaluate the resistivity and conductivity of electrocatalysts. Figure 7c shows the Nyquist plots of all samples measured at open circuit voltage, where the diameter of the semicircle reflects the charge transfer resistance (*R*_ct_) between electrocatalyst and electrolyte. It can clearly be seen from the graph that the semicircle diameter of Co_3_O_4_-RuO_2_-10 is the smallest compared to the other samples. The fitted *R*_ct_ value of Co_3_O_4_-RuO_2_-10 is 2.42 Ω, which is much lower than that of the Co_3_O_4_, Co_3_O_4_-RuO_2_-5, and Co_3_O_4_-RuO_2_-15 samples, with *R*_ct_ values of 8.70, 4.03, and 5.26 Ω, respectively (Table 1). The results suggest that Co_3_O_4_-RuO_2_-10 has a stronger charge transfer ability and faster reaction rate. This is consistent with the test results of LSV and Tafel curves. The results show that if too low or too high a content of Ru^3+^ added, the optimal catalytic performance will not be obtained. Only introducing an appropriate amount of the Ru element can produce the best OER performance.

In addition, CV measurements were conducted at different scan rates in the non-Faraday region (0–0.1 V vs. Hg/HgO), and *C*_dl_ values were obtained by linear-fitting the difference in current density with scan rates to evaluate the electrochemical surface area (ECSA) and its corresponding intrinsic activity. Figure S3a–d show the corresponding CV curves of Co_3_O_4_, Co_3_O_4_-RuO_2_-5, Co_3_O_4_-RuO_2_-10, and Co_3_O_4_-RuO_2_-15 at different scan rates (20, 40, 60, 80, and 100 mV s^−1^). Figure 7d shows the linear fit between the difference in current density and scan rate. It can be concluded that Co_3_O_4_-RuO_2_-10 has the highest slope, indicating that it has the highest *C*_dl_ value of 2.77 mF cm^−2^. The *C*_dl_ values of Co_3_O_4_, Co_3_O_4_-RuO_2_-5, and Co_3_O_4_-RuO_2_-15 are 0.29, 1.74, and 1.10 mF cm^−2^, respectively. This indirectly proves that the Co_3_O_4_-RuO_2_-10 sample has the highest ECSA and OER electrocatalytic activity.

**Figure 8:**
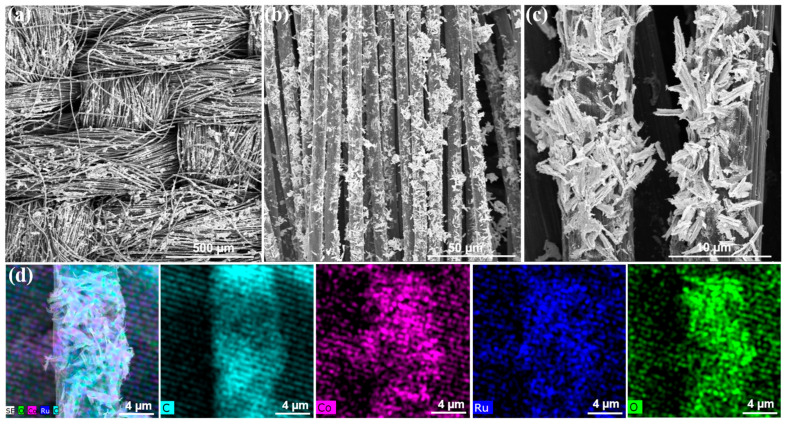
(**a**–**c**) SEM images of Co_3_O_4_-RuO_2_-10/CC; (**d**) EDX mapping diagram of optimal Co_3_O_4_-RuO_2_-10/CC sample.

**Figure 9:**
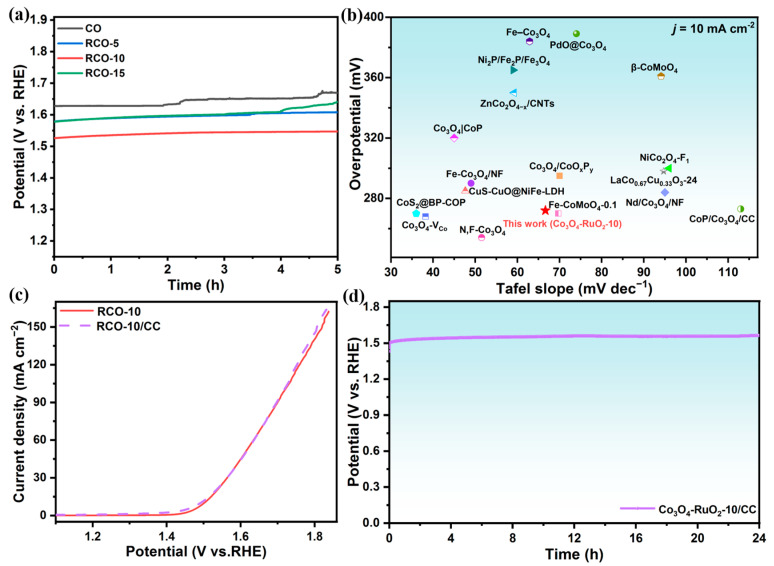
(**a**) Stability tests of Co_3_O_4_, Co_3_O_4_-RuO_2_-5, Co_3_O_4_-RuO_2_-10, and Co_3_O_4_-RuO_2_-15 on RDE for 5 h at 10 mAcm^−2^; (**b**) comparison of the overpotentials and Tafel slopes of various electrocatalysts; (**c**) LSV profiles of Co_3_O_4_-RuO_2_-10 and Co_3_O_4_-RuO_2_-10/CC without iR compensation; (**d**) chronopotentiometric curve for Co_3_O_4_-RuO_2_-10/CC self-supporting electrode at 10 mAcm^−2^.

In order to evaluate the long-time stability of Co_3_O_4_-RuO_2_-10 sample with the best electrocatalytic performance, the RuO_2_-Co_3_O_4_-10/CC heterostructure was also prepared in this study for large-scale application as an integrated electrode for the electrocatalytic OER over a long period (Figures S4–S6). Figure 8 confirms that the smaller and thinner nanosheet assemblies are firmly grown on carbon cloth fibers, which helped to guarantee the robust durability of Co_3_O_4_-RuO_2_-10/CC during the OER tests. Firstly, the stability of all powder samples was evaluated using the constant current method (*j* = 10 mA cm^−2^) on the RDE. Figure 9a shows the potential changes in Co_3_O_4_, Co_3_O_4_-RuO_2_-5, Co_3_O_4_-RuO_2_-10, and Co_3_O_4_-RuO_2_-15 within 5 h. There was a significant jump in the potential of Co_3_O_4_, Co_3_O_4_-RuO_2_-5, and Co_3_O_4_-RuO_2_-15 during the testing process, which may be due to the detachment of the catalyst film on the RDE surface. Under the same conditions, only Co_3_O_4_-RuO_2_-10 was less affected by bubbles during stability testing at 1600 r.p.m., and the catalyst film on the electrode surface did not detach. The amplitude of potential change was the smallest, demonstrating its good stability. In addition, the *η*_10_ and Tafel slope of Co_3_O_4_-RuO_2_-10 (RCO-10) and other transition-metal electrocatalysts for the OER are also compared in Figure 9b. It is noteworthy that RuO_2_-Co_3_O_4_-10 exhibits superior OER performance and durability, which are comparable to the related transition-metal-based electrocatalysts recently reported, and better than that of pure RuO_2_ (317 mV) and commercial IrO_2_ catalysts (374 mV) (details in Table S1). Furthermore, Figure 9c and Figure S7 demonstrate that Co_3_O_4_-RuO_2_-10/CC shows a small amplitude increase in activity compared to the Co_3_O_4_-RuO_2_-10 powder catalyst fixed using Nafion, with a lower overpotential of 262 mV at 10 mA cm^−2^, a small Tafel slope and a minimal charge transfer resistance. Figure 9d shows the chronopotentiometric (CP) responses of Co_3_O_4_-RuO_2_-10/CC at 10 mA cm^−2^, which display a very low degradation and almost no detachment of the catalyst during the testing process. In addition, the morphology, crystal structure, and composition of the Co_3_O_4_-RuO_2_-10/CC after the stability test are further characterized in Figure S8a–e. In Figure S8a–c, there are no obvious changes in the morphology of Co_3_O_4_-RuO_2_-10/CC. In Figure S8d, the peak intensity at 35.32° for RuO_2_ shows a slight decrease, which is also supported by the slight reduction in the percentage of Ru atoms in Figure S8e. Therefore, the self-supported Co_3_O_4_-RuO_2_-10/CC helps to maintain the high activity and stability of the catalyst.

## 4. Conclusions

In this work, a simple solvothermal method was used to prepare Co-MOFs. Then, the Ru element was successfully introduced into the framework through an ion exchange process. Finally, RuCo-MOFs with different Ru contents were calcined at 350 °C to successfully synthesize a series of RuO_2_-Co_3_O_4_ composite samples. A series of phase characterizations were carried out on the optimal material, Co_3_O_4_-RuO_2_-10, and its robust layer structure and large BET specific surface area were key factors in its excellent catalytic performance. The successfully introduced Ru element contributes more electrons to the O element due to its high electronegativity, thus enhancing its electron transfer ability and improving its electrocatalytic activity by adjusting the electronic structure of the catalyst surface. Therefore, compared with the same series of catalysts, Co_3_O_4_-RuO_2_-10 exhibits a lower overpotential (*η*_10_ = 272 mV) and smaller Tafel slope (64.64 mV dec^−1^). Meanwhile, the stability and catalytic performance were substantially enhanced by growing the Co-MOF precursor on carbon cloth and further transforming it into a Co_3_O_4_-RuO_2_-10/CC self-supported electrode. This work may provide a method for the design of transition-metal oxide heterostructures and their application in water electrolysis.

## Figures and Tables

**Table 1:** Comparison of OER performance of series samples in alkaline medium.

Catalysts	Overpotential (mV) (*j* = 10 mA cm^−2^)	Tafel Slope (mV dec^−1^)	*R*_ct_ (Ω)
CO	399	77.10	8.70
RCO-5	331	76.73	4.03
RCO-10	272	66.64	2.42
RCO-15	322	71.98	5.26

## Data Availability

The original contributions presented in this study are included in the article/Supplementary Material. Further inquiries can be directed to the corresponding author.

## Supplementary Materials

The following supporting information can be downloaded at https://www.mdpi.com/article/10.3390/nano15171356/s1, Figure S1: XRD pattern of Co-MOF precursor; Figure S2: EDX of Co_3_O_4_ and Co_3_O_4_-RuO_2_ series samples; Figure S3: (a) CV curves of Co_3_O_4_, (b) Co_3_O_4_-RuO_2_-5, (c) Co_3_O_4_-RuO_2_-10, and (d) Co_3_O_4_-RuO_2_-15 at different scan rates (20–100 mV s^−1^); Figure S4: Digital photos of Co-MOF/CC, RuCo-MOF-10/CC, and Co_3_O_4_-RuO_2_-10/CC; Figure S5: LSV polarization curve of Co_3_O_4_-RuO_2_-10 on RDE with 90% iR-compensation; Figure S6: (a) XRD pattern and (b) EDX of Co_3_O_4_-RuO_2_-10/CC; Figure S7: Tafel slope and Nyquist plot of Co_3_O_4_-RuO_2_-10/CC; Figure S8: (a–c) SEM images, (d) XRD pattern and (e) EDX of Co_3_O_4_-RuO_2_-10/CC after stability tests; Table S1: Comparison of the overpotentials at a current density of 10 mAcm^−2^ for OER in alkaline electrolyte with the reported transition metal based electrocatalysts.
