# Supplementary material for: Synthesis of RuO2-Co3O4 Composite for Efficient Electrocatalytic Oxygen Evolution Reaction
Source: Nanomaterials (Basel). 2025 Sep 3;15(17):1356. doi: 10.3390/nano15171356 (PMC12429896; doi:10.3390/nano15171356)
Supplement: Supplementary file 1 [file nanomaterials-15-01356-s001.zip › nanomaterials-3804530-supplementary.pdf]

Supporting information

Synthesis of RuO_2-Co_3O_4 Composite for Efficient Electrocatalytic Oxygen Evolution Reaction

Jingchao Zhang,[a]* Yingping Bu,[a,b] Jia Hao,[a] Wenjun Zhang,[a] Yao Xiao,[a] Naihui Zhao,[a] Renchun Zhang,[a] Daojun Zhang[a]*

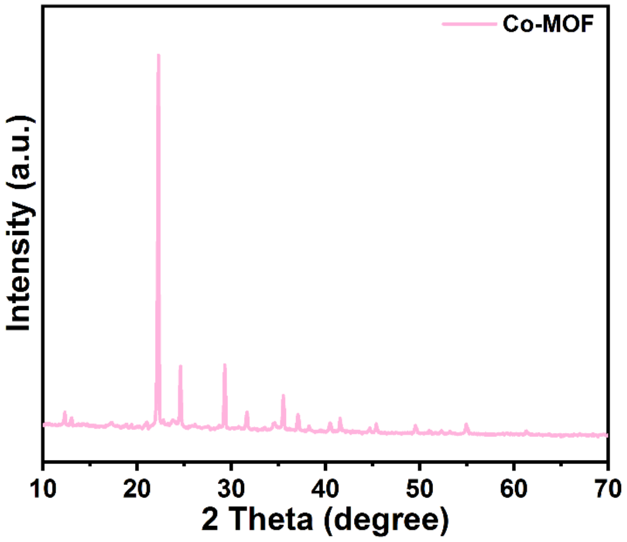

Figure S1 XRD pattern of Co-MOF precursor.

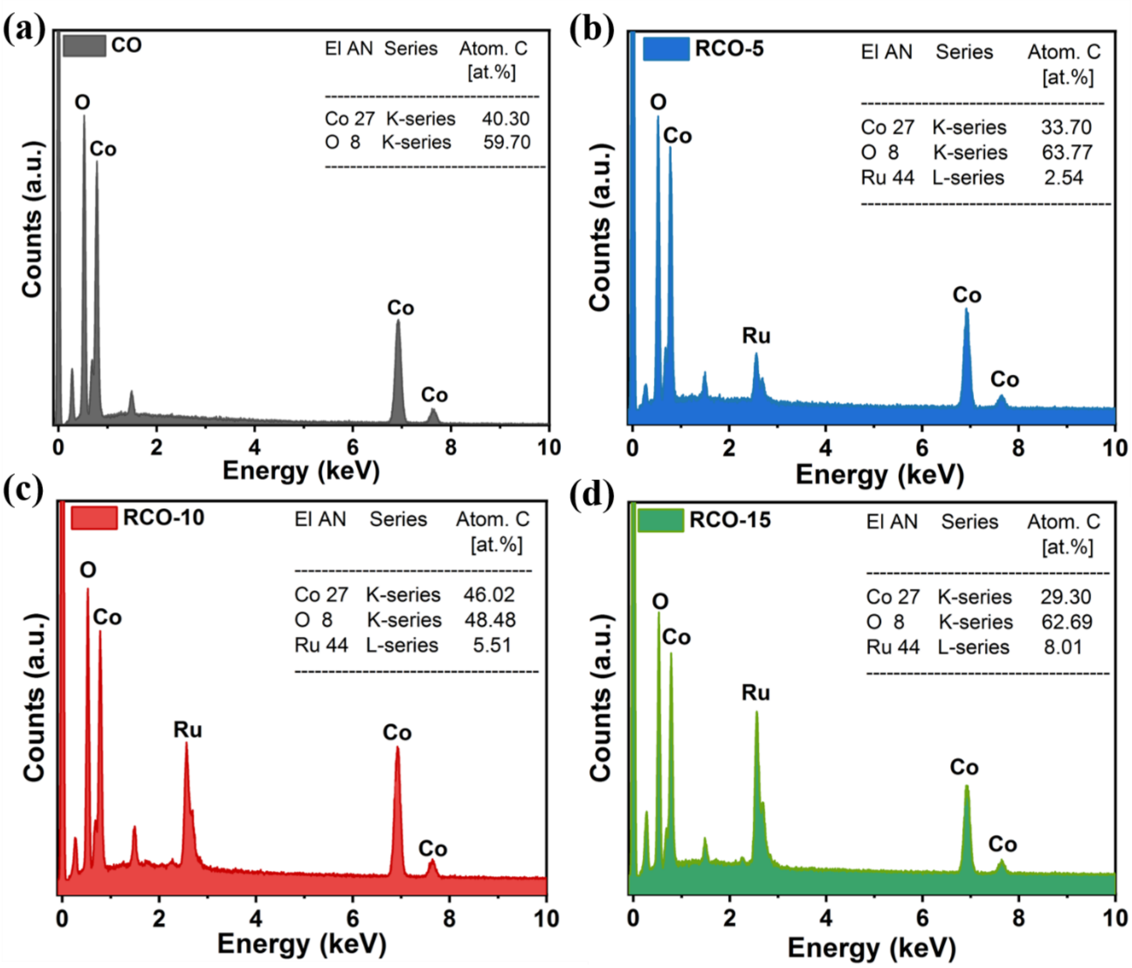

Figure S2 EDX of Co_3O_4 and Co_3O_4-RuO_2 series samples.

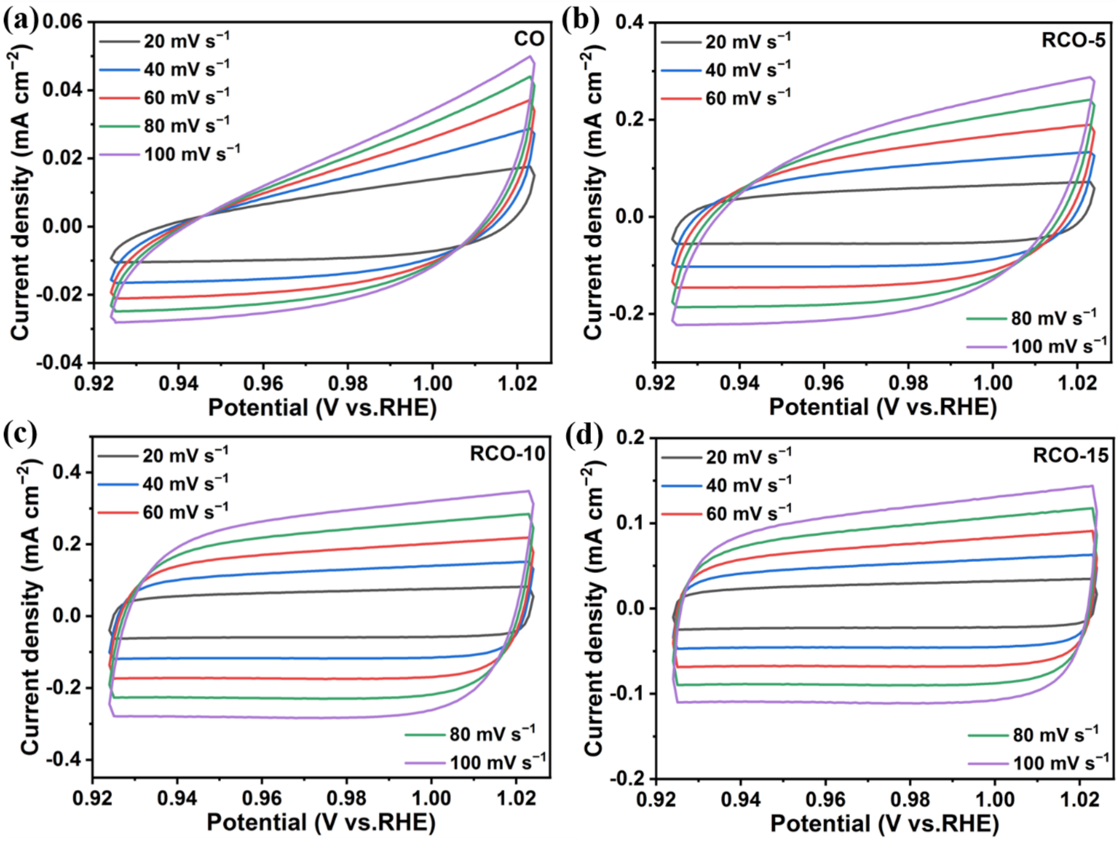

Figure S3 (a) CV curves of Co_3O_4, (b) Co_3O_4-RuO_2 -5, (c) Co_3O_4-RuO_2 -10, and (d) Co_3O_4-RuO_2 -15 at different scan rates (20~100 mV s^{-1}).

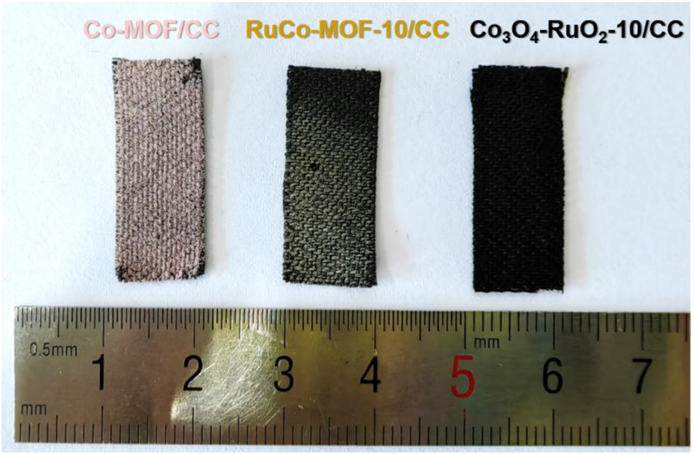

Figure S4 Digital photos of Co-MOF/CC, RuCo-MOF-10/CC, and Co_3O_4-RuO_2-10/CC.

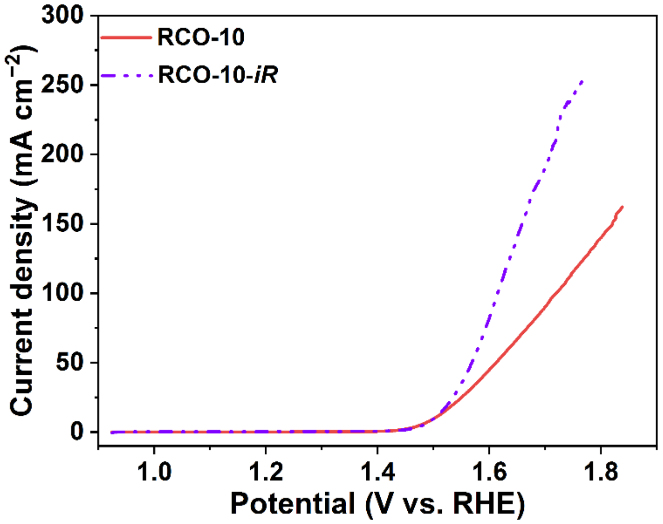

Figure S5 LSV polarization curves of Co_3O_4-RuO_2-10 on RDE with 90% iR-compensation and without iR-compensation.

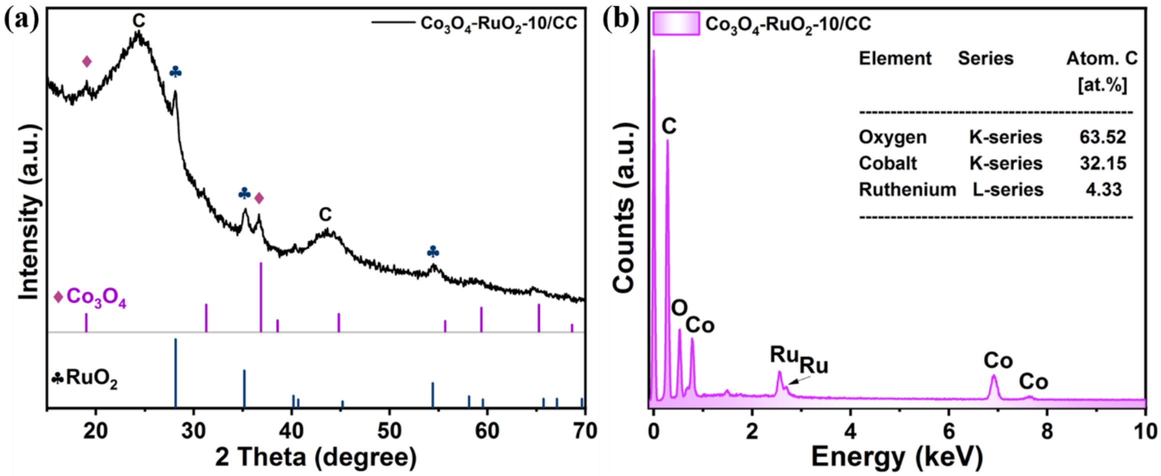

Figure S6 (a) XRD pattern and (b) EDX of Co_3O_4-RuO_2-10/CC.

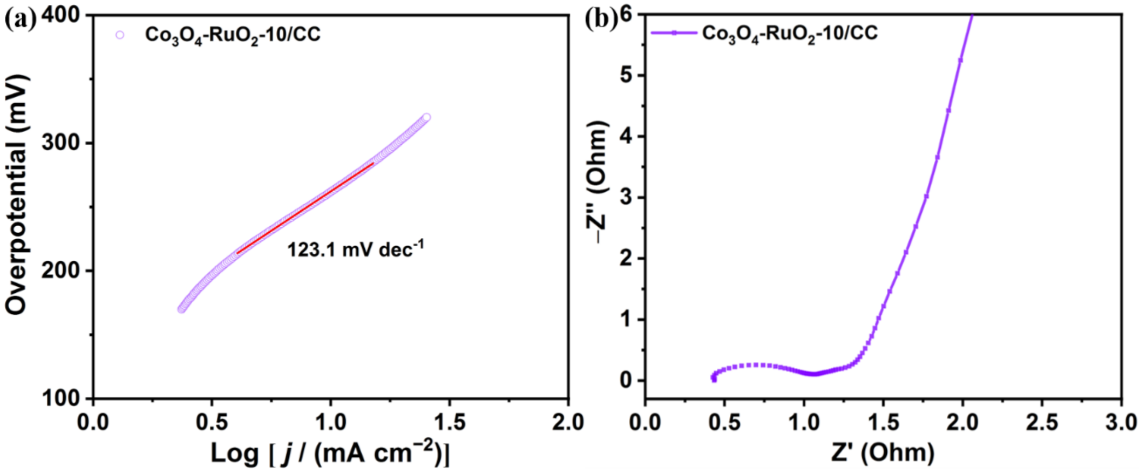

Figure S7 (a) Tafel slope and (b) Nyquist plot of Co_3O_4-RuO_2-10/CC.

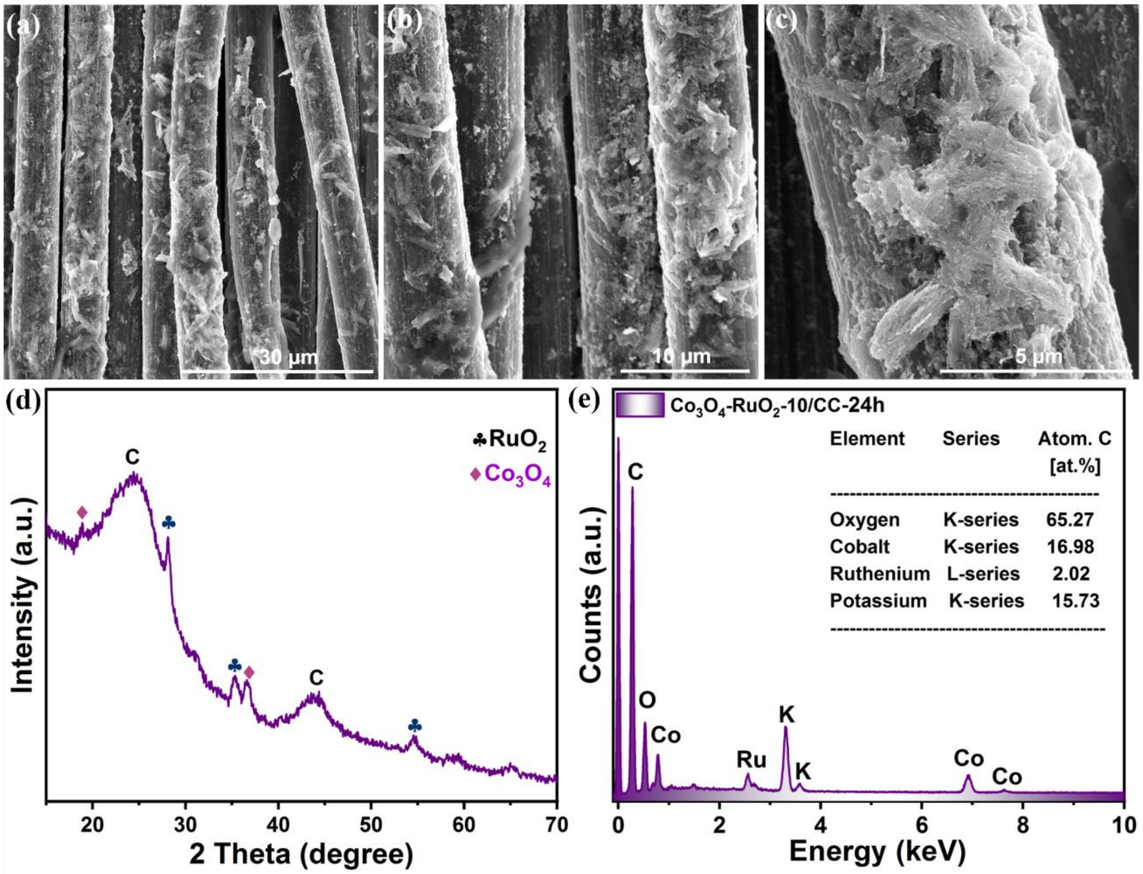

Figure S8 (a-c) SEM images, (d) XRD pattern and (e) EDX of Co_3O_4-RuO_2-10/CC after stability tests.

Table S1 Comparison of the overpotentials at a current density of 10 $mAcm^{-2}$ for OER in alkaline electrolyte with the reported transition metal based electrocatalysts.

**Catalyst**	η_{10} **(mV)**	**Tafel slope (mV dec^{-1})**	**Durability** (10 mA cm^{-2})	**Ref.**
Co_3O_4-RuO_2-10 (RCO-10)	272	66.64	5 h	This work
Co_3O_4-RuO_2-10/CC (RCO-10/CC)	262	123.10	24 h	This work
$MgCo_2O_4$-500	283	66	1.51 V (vs RHE), 12 h	*ACS Sustainable Chem. Eng.* 2023, 11, 744−750.
Co_3O_4|CoP	320	45	18 h	*ACS Appl. Nano Mater.* 2022, 5, 9150–9158.
Mn-RuO_2<br>RuO_2<br>IrO_2	270<br>317<br>374	45.8<br>/<br>/	20 h<br>/<br>7.5 h	*J. Am. Chem. Soc.* 2022, 144, 2694−2704
CoP/Co_3O_4/CC	273	113	30 h	*ACS Appl. Nano Mater.* 2025, 8, 4899–4910
Ni_2P/Fe_2P/Fe_3O_4	365	59	28800 s	*Chem Asian J.* 2019, 14, 2744–2750.
Co_3O_4/CoO_xP_y	295	70	30000 s	*ChemNanoMat* 2019, 5, 1390–1397.
Vo-Cubic-Co_3O_4	375	58.2	25 h	*ACS Energy Lett.* 2024, 9, 2182–2192.
Nd/Co_3O_4/NF	284	95	/	*J. Energy Chem.* 2025,106, 142–150.
Fe–Co_3O_4	384	62.9	60 h	*ACS Catal.* 2024,14, 5888–5897.
Cu-F-Co_3O_4-0.7	290	111	>100 h	*J.Colloid Interf.Sci.* 2025, 690, 137288.
Fe-Co_3O_4/NF	290	49	200 h	*Molecules* 2025, 30, 1046.
CoS_2@BP-COP	270	36	24 h	*Chem Asian J.* 2021, 16, 3102–3106
N, F-Co_3O_4	254	51.5	20 mA cm^{-2}, 240 h	*Adv. Mater.* 2025, 2501381.
$NiCo_2O_4$-F_1	300	96	10 mA cm^{-2}, 36 h;<br>50 mA cm^{-2}, 36 h;<br>100 mA cm^{-2}, 36 h.	*Adv. Mater.* 2025, 2418058.
$ZnCo_2O_{4-x}$/CNTs	350	59.2	/	*Angew.Chem.Int.Ed.*2023,62, e20230140.
PdO@Co_3O_4	389	74	2 h	*Energy Fuels* 2022, 36, 12719−12728.
Co_3O_4-V_{Co}	268	38.2	1.42 V vs RHE (onset potential), 4 h	*J.Am.Chem.Soc.* 2023, 145, 2271–2281.
Fe-$CoMoO_4$-0.1	270	69.7	/	*Inorg.Chem.* 2025, 64,

Fe-$CoMoO_4$-0.2	276	66.3	40 h	2508–2517.
RuO_2	312	196.1	40 h	
$LaCo_{0.67}Cu_{0.33}O_3$-24	298	94.7	25 h	*Inorg.Chem*. 2025, 64, 10533–10541.
β-$CoMoO_4$	361	94.10	/	*ACS Catal*. 2025, 15, 11958−11969.
CuS–CuO@NiFe-LDH	285	47.65	15 h	*New J. Chem*. 2025, 49, 10832−10840.
RuO_2	338	/	~6 h	
Co_3O_4/CC	357	283	30 h	*Int. J. Hydrogen Energy* 2024, 52, 482−493.
